# Mobility paradoxes: disruptors, benefits, and agency among mobile female sex workers living with HIV in the Dominican Republic and Tanzania

**DOI:** 10.1186/s44263-023-00032-3

**Published:** 2024-01-16

**Authors:** Maria De Jesus, Zoé Hendrickson, Julia Rivara, Clare Barrington, Yeycy Donastorg, Martha Perez, Hoisex Gomez, Jessie Mbwambo, Samuel Likindikoki, Deanna Kerrigan

**Affiliations:** 1 Department of Environment, Development, and Health, School of International Service, American University, Washington, DC USA; 2 Center on Health, Risk, and Society, American University, Washington, DC USA; 3 Johns Hopkins Center for Communication Programs, Johns Hopkins Bloomberg School of Public Health, Baltimore, MD USA; 4 Department of Health Behavior, Gillings School of Global Public Health, University of North Carolina at Chapel Hill, Chapel Hill, NC USA; 5 Instituto Dermatológico y Cirugía de La Piel, Santo Domingo, Dominican Republic; 6 Muhimibili University of Health and Allied Sciences, Dar Es Salaam, Tanzania; 7 Department of Prevention and Community Health, Milken Institute School of Public Health, George Washington University, Washington, DC USA

**Keywords:** Sex work, Mobility, Female sex workers, Dominican Republic, Tanzania, HIV, Social determinants of health

## Abstract

**Background:**

Mobility is a key social determinant of health for female sex workers (FSWs). While extant research has focused on the adverse effects of mobility for FSWs, there are very few studies that have examined the multiple ways in which mobility may impact the lives of these mobile women from their perspective. This qualitative study aims to fill this gap by exploring how mobility impacts the lives, livelihoods, and HIV care and treatment from the perspectives of women living with HIV in two epidemic settings, the Dominican Republic and Tanzania.

**Methods:**

We conducted two rounds of in-depth interviews with 12 Dominican women and 12 Tanzanian women who were 18 years plus, had an HIV-positive diagnosis, and reported having exchanged sex for money in the last month. We utilized thematic analysis with a focus on intra- and intercomparisons to observe patterns within and across the two contexts.

**Results:**

We identified a salient pattern of three thematic “mobility paradoxes,” which related to both disruptors and benefits of sex work mobility: (1) uncertainty versus autonomy: while sex work mobility often took place in contexts of vulnerability, which often led to women experiencing violence, it simultaneously benefitted women by allowing them to choose where they stayed and make plans on their terms; (2) financial insecurity versus profitability: although participants sometimes made less money than expected when they traveled for sex work, there was a powerful economic benefit for mobile women as it increased their likelihood of profitability; and (3) disorder and interruptions versus strategy and social support in HIV care and treatment: participants reported that they were sometimes inconsistent with their HIV appointments and medications when they traveled for sex work. On the other hand, mobility contributed to participants often becoming more strategic and creative in their HIV care and treatment and relying on one another for support.

**Conclusions:**

Findings indicate that sex work mobility is a nuanced, complex, and paradoxical phenomenon. Implications include the development of strengths-based and community empowerment mobile health initiatives tailored to mitigate disruptors of mobility while maximizing benefits for this population.

**Supplementary Information:**

The online version contains supplementary material available at 10.1186/s44263-023-00032-3.

## Background

Globally, female sex workers (FSWs) are 30 times more likely to be HIV positive than women who are not sex workers [1]. Social determinants such as intersectional stigma, discrimination and violence, poverty, and gender inequality heighten vulnerability to HIV infection as well as inhibit optimal care and treatment outcomes among this population [1]. The current study is guided by a social determinants framework, which begins with the assumption that certain groups of individuals experience rates of a given disease or health outcome differentially, due to structural factors, and their social status and circumstances [2].

One such circumstance is mobility, a key social determinant of health that impacts HIV outcomes among FSWs [3–6]. There is no consistent definition of mobility in the literature, and it is often used interchangeably with the term “migrant.” Bell and Ward [7] refer to mobility as any form of movement that is not permanent. Mobility has many dimensions, including the frequency, distance, transit mode, reason, seasonality, and duration of travels [7, 8]. In this article, we define mobility as the voluntary and temporary movement of individuals to specific location(s), who then return to their home after a specific amount of time. *Sex work* mobility refers to traveling for the purposes of exchanging sex for money in a place(s) away from one’s home (e.g., in another district or region) temporarily [9].For many of the women, sex work mobility is the main strategy to earn money to support themselves and their families [10].

A lack of a clear definition for “mobility” and studies that differentiate between mobile and nonmobile FSWs make it challenging to obtain population size estimates for mobile women. In addition, an exact global figure for percentage of women who engage in mobile sex work is difficult to obtain as there exists great variation among populations and geographical settings. For example, Hendrickson et al. found that 33% of FSWs in Iringa, Tanzania, were mobile outside of their district or region only, while 12% were mobile for sex work both within and outside their region [11]. Another study revealed that approximately 11% of the FSWs in Vancouver, Canada, were mobile [12]. In Saggurti et al.’s study [13] in four regions of India denoted for their seasonal workers, the population of mobile women ranged anywhere from 30 to 65% of the sample depending on the age, duration, and number of moves. In Zimbabwe, mobility among FSWs reached up to 59% in 2016 [14]. In sum, empirical data on population size estimates of these mobile women are patchy.

Both Santo Domingo in the Dominican Republic (DR) and Iringa in Tanzania are distinct geographic settings where there exists a heightened risk for HIV infection, particularly among the key population of FSWs. The prevalence for HIV is 3.7% and 15.4% among FSWs in the DR and Tanzania, respectively, which is significantly higher than the national HIV prevalence in each country (0.9% in the DR and 4.7% in Tanzania) [15]. Our research group has conducted extensive formative and intervention research [2, 16] to assess the landscape of social determinants of HIV among FSWs in these two epidemic settings.

The geospatial contexts of sex work mobility in the DR and Tanzania differ substantially. Santo Domingo is the largest city and capital of the Dominican Republic, with a population of 2.2 million [17]. The city serves as an important seaport along the Ozama River, and it is an economic hub for the country. Like other Caribbean islands, the DR with its mild tropical climate is a tourist destination for many travelers, particularly from North America and Europe, who vacation in beach towns. FSWs often travel from the capital to these more distant beach towns to meet tourists who are either longtime or new clients. Others travel to neighboring or distant smaller *pueblos* (towns) where they meet their regular clientele comprised mostly of local and seasonal workers.

In contrast, Iringa is in the Southern Highlands of Tanzania. The administrative capital of the region is Iringa city, with a population of approximately 112,000 [18]. Agricultural production and transport are a key source of the local economy [19]. Similar to other settings in the sub-Saharan region, mobility in Iringa is influenced by the Tanzanian-Zambian (Tan-Zam) highway that cuts through the geographical area [19]. The movement of truck drivers and the seasonal migration of residents and those from outside the region for agricultural work have made mobility an important aspect of life. For FSWs, the Tan-Zam highway is a source of clients and plays a role in their mobility [19].

The current study drew on existing cohorts in Iringa, Tanzania, and Santo Domingo, DR [2]. While the geospatial contexts of sex work mobility are different in these two geographic and epidemic settings, the drivers and conditions of mobility are similar among women engaged in sex work across these two sites. Sex work mobility is a key livelihood strategy for the mobile FSWs, many of whom are mothers under the age of 40. In both settings, these women strategically move to areas where there is greater market demand, traveling to places where they can earn higher wages [4, 12, 20]. Sex work mobility is, therefore, a form of capital and economic power, which allows FSWs to increase their income beyond what it would have been if they stayed in one location [2, 10, 12, 21].

Several studies have examined the effect of mobility on HIV outcomes and access and adherence to HIV-related services among mobile populations [3, 12, 20]. A recent systematic review on the effect of mobility on adherence to HIV-related services demonstrated that mobility is associated with increased odds of poor linkage to care and antiretroviral therapy (ART) interruption for these women [20]. In addition, Hendrickson et al.’s study [11] demonstrated that mobile FSWs had 1.9 times greater odds of reporting recent gender-based violence (GBV) compared with their nonmobile counterparts and a 2.5 times higher relative risk for recent experience of severe GBV relative to no recent GBV. Similarly, several other studies in other geographic locations reported that mobility for sex work was associated with an increase in workplace sexual and physical violence [3, 12, 20]. While considerable focus has been placed on the negative effects of mobility for these women, there have been very few in-depth qualitative studies to date on the perceptions and experiences of this population, including those living with HIV to shed light on the multiple ways in which mobility impacts their lives [22, 23]. The purpose of the current study is to fill this gap by exploring in-depth how mobility influences the lives, livelihoods, and HIV care and treatment experiences of Dominican and Tanzanian FSWs living with HIV.

## Methods

This study draws on in-depth 90-min interviews conducted with a subcohort of FSW living with HIV established in 2017 using a stratified, purposeful sample of FSWs who participated in the larger parent study, titled *Stigma, cohesion, and HIV outcomes among vulnerable women across epidemic settings* (R01MH110158) [16]. This longitudinal mixed-methods research study followed 400 FSWs living with HIV in Santo Domingo, DR) and Iringa, Tanzania, to examine the social determinants of HIV outcomes among FSWs living with HIV during the period of 2016–2021.

In the DR, a cohort of 200 FSWs living with HIV in the greater Santo Domingo was established in 2011 as part of an implementation science project called *Abriendo Puertas* (Opening Doors) focused on improving HIV care and treatment outcomes (2). Initial recruitment took place predominately by FSW *navegadoras* (peer navigators) from our sex worker community partner organization in the DR, *Movimiento de Mujeres Unidas* (MODEMU), who identified and contacted potential participants. The approach was complemented by recruitment from clinics and other key informants, as well as participants themselves.

In Tanzania, a cohort of 200 FSWs in Iringa in 2015 was recruited using time-location sampling, as part of a randomized trial of a community-driven intervention called *Project Shikamana* (Let’s Stick Together), which sought to reduce HIV incidence and improve care continuum outcomes [2]. Quantitative surveys were complemented by two rounds of qualitative interviews. In the first round of interviews, mobility emerged organically. Consequently, in the second round of interviews, a mobility module was included to explore in-depth participants’ recent mobility experiences.

This study focuses on the qualitative data from the second round of interviews with a subset of cohort participants in each country (*n* = 12 Dominicans and *n* = 12 Tanzanians), who had reported traveling outside of Santo Domingo/Iringa in the last 6 months in the quantitative survey [2]. In both settings, inclusion criteria included women at least 18 years of age, with a confirmed HIV-positive diagnosis using a single rapid test, and who reported having exchanged sex for money in the last month prior to their enrollment in the study.

Follow-up interviews were conducted in Santo Domingo and Iringa between December 2019 and March 2020. Four trained female interviewers who had developed trust and rapport with the participants conducted the in-depth interviews in either Spanish (DR) or Swahili (Tanzania). We used a semi-structured interview guide (see Additional file 1) common to both settings with additional country-specific questions, including a module specific to mobility. These questions were intended to purposefully probe regarding the participants’ mobility to enrich our understanding of how they conveyed meaning to their experiences with mobility. We also asked about details related to their preparations before they traveled, the experiences during their travels and at their destination, and their social relationships and HIV care-seeking and ART adherence when they travel. Sample mobility-related questions included a free-listing activity asking participants to think about all the different places they typically go in and outside of Iringa/Santo Domingo and an in-depth exploration of a typical trip they have made outside of Iringa/Santo Domingo. Interviewers probed for information on where they went, for how long, preparations made before travel, details about the trip including how they got there, difficulties encountered, purpose of travel, social relationships at place of destination, types of support received there, cell phone use, adherence to HIV care and treatment, and recommendations for future programs unique to this population. A COnsolidated criteria for REporting Qualitative research (COREQ) checklist is provided (see Additional file 2).

All interviews were audio-recorded and transcribed verbatim to facilitate analysis [24]. Interviews from Tanzania were translated from Swahili to English for analysis, while interviews from the DR were analyzed in Spanish. The thematic analysis approach was an iterative, data-driven, and inductive process [25, 26]. Two coders used memo writing throughout the coding process to document emerging themes and observations about the data and our thoughts about the significance and relationships of codes to one another, as well as to assist with data reduction and interpretation [25, 26].

**Fig. 1:**
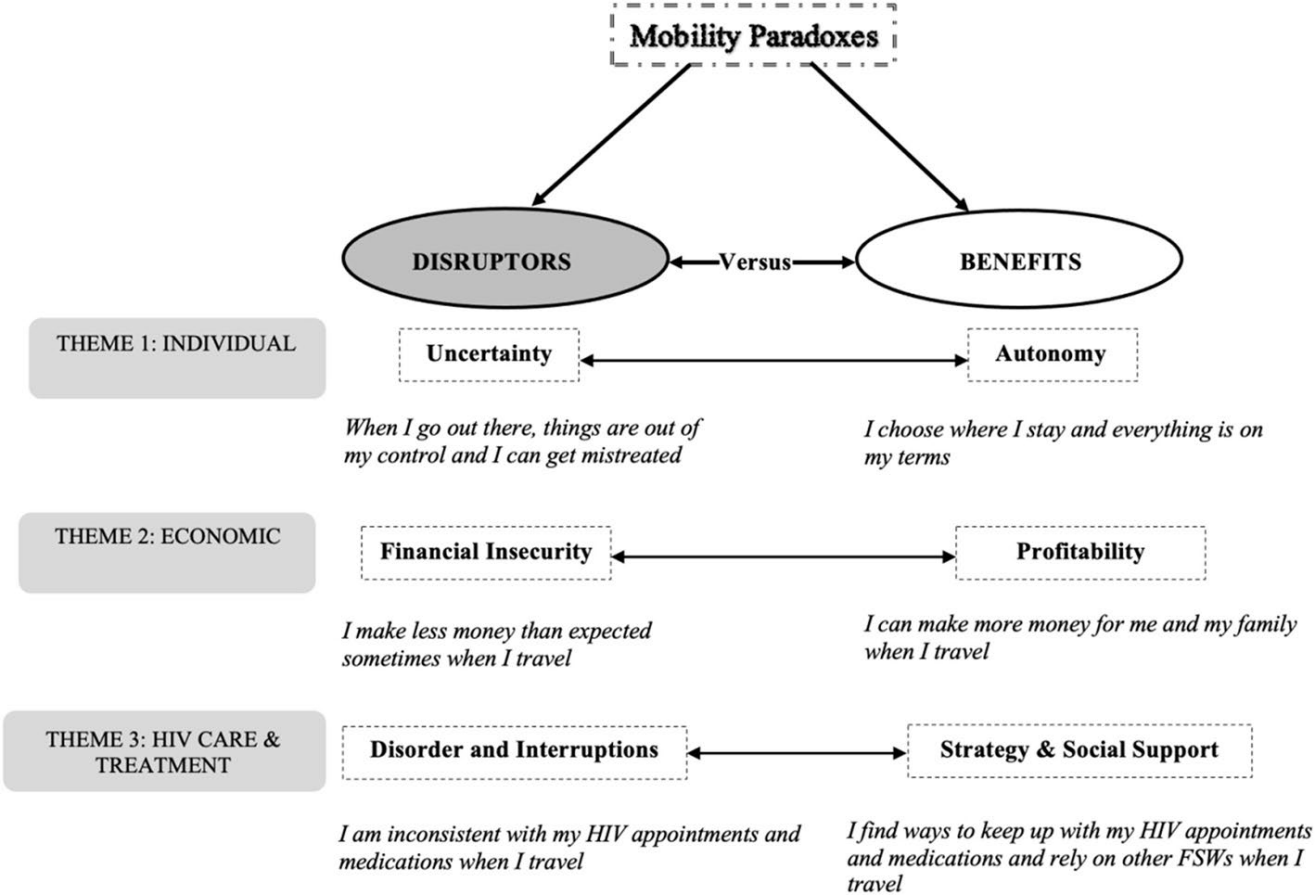
A coding framework of the study

Our memos also facilitated integration of themes over two different time periods (initial and follow-up interviews) to gain a deeper, more nuanced, and contextualized understanding of mobility and how the dynamic phenomenon played out in the lives of the participants. We used constant intra- and intercomparisons to look for patterns within and across the two contexts and to reduce the number of codes [25, 26]. We developed a codebook based on the themes emerging from the data. Using our memos, we then synthesized the coding output across key domains, identified major themes, and listed in vivo codes under major themes. The in vivo codes evoked the essence of the participants’ experiences and preserved the affective tone of their words. Major themes under which codes were arranged included the following: uncertainty versus autonomy, financial insecurity versus profitability, and disorder and interruptions versus strategy and social support. These major categories were subsumed under a meta-theme, *mobility paradoxes*. Figure 1 provides a coding framework of the study.

## Results

As shown in Table 1, participants across geographic settings ranged from 20 to 52 years old. On average, the Dominican participants were older (mean = 40) compared to the Tanzanian participants (mean = 28). Most of the participants in Tanzania and approximately half of the Dominican participants were single or not currently living with a partner. All participants in the sample had children, with a mean number of 2, and all lived in either Santo Domingo (capital of the DR) or Iringa (city in Central Tanzania), respectively. Education levels were low with all the participants from the Dominican Republic having primary-level education and those from Tanzania having either primary-level education (7) or some secondary schooling (5). The participants in Tanzania had been engaging in sex work for an average of 8 years mostly in modern bars, while Dominican participants had been engaging in sex work for a much longer duration (21 years) in different venues (i.e., on the street or in a sex establishment) or independently. The frequency of sex work varied across both settings, and the length of stay at their travel destination ranged from 1 day to 3 months.

**Table 1:** Sociodemographic characteristics among mobile female sex workers living with HIV in the Dominican Republic and Tanzania

Variables	Dominican Republic (*n* = 12)	Tanzania (*n* = 12)
**Age**: Mean in years (range)	40 (21–53)	28 (20–48)
**Civil/marital status**		
Single/not currently living with a sex partner	5	9
Living with sex partner	6	1
Married and living with sex partner	0	2
Widowed	1	0
**Education level**		
0–8th grade (primary education)	12	7
Any secondary school	0	5
**Has any children**	12	12
**Number of children**: Mean number (range)	2 (1–3)	2 (1–5)
**Current residence**		
Santo Domingo or Iringa	12	12
**Type of sex work venue**		
Street	6	0
Sex establishment or independent	6	0
Modern bar	0	11
Other	0	1
**Years in sex work**: Mean (range)	21 (8–36)	8 (1–19)
**Frequency of sex work**		
Once a day or more	1	1
A few times a week	4	7
About once a week	2	0
A couple times a month	5	3
Other	0	1
**Duration of stay at destination** (range)	1 day-3 months	1 day-3 months

The meta-theme of the study focuses on “mobility paradoxes,” which are characterized as both disruptors and benefits in the lives of mobile Dominican and Tanzanian participants. The three major themes are as follows: (1) individual disruptors and benefits: uncertainty versus autonomy; (2) economic disruptors and benefits: financial insecurity versus profitability; and (3) HIV care and treatment disruptors and benefits: disorder and interruptions versus strategy and social support.

These themes reflect the participants’ nuanced and emic understandings of the impact of sex work mobility on their lives, livelihoods, and HIV care and treatment. The findings are reported below, and pseudonyms are used.

### Theme 1: Individual disruptors and benefits—uncertainty versus autonomy

Sex work mobility often took place in contexts of vulnerability and uncertainty, which often led to women experiencing insecurity. The first in vivo code, *When I go out there, things are out of my control and I can get mistreated*, referred to a disruptor of mobility for women living with HIV. Participants from both settings reported incidents of violence, exploitation, and human right abuses by police, clients, and bar/hotel owners. A Dominican participant described the abuse perpetrated by the police against mobile women and the sense of disempowerment that these women confront when they travel. In this case, she had gone to the beach town located in the eastern region of the island and heavily populated by tourists:

In Samaná where I travel, the situation has been difficult. Some businesses have closed, and the police have been bothering us a lot, especially now because the government has closed a few of the businesses where we were offering our sex services. The women go out more on the streets and the police harass them. Many of them have been imprisoned. There is nothing they can do. -Dominican Republic, age 25

Another participant shared a story of violence she experienced at the hands of a client (who in this case was also a police officer) and how she felt alone and unsupported. She had traveled to a beach town in Puerto Plata, a northern province of the Dominican Republic, known for its sandy beaches:

I had a client who hit me when I traveled to Sosúa. He offered me money to go with him and I went out with him […] We were having sex and he ejaculated too fast and he wanted me to give him back his money, so that’s when the argument started and then he hit me. I wanted to go to the police, but I was too scared […] because he was a police officer too and I knew that he could talk and create problems for me. I was a woman who didn’t have support from another person or anything. -Dominican Republic, age 30 .

In both narratives, the participants were victims of violence and had no one to protect or support them. Several participants from the Dominican Republic and Tanzania, however, mentioned taking precautions whenever possible to protect themselves from potential violence from a client when traveling. For example, in the narrative below, a Tanzanian participant shared her destination with fellow FSWs before leaving and jotted down the client’s license plate. She referred to these precautions as “preparations,” but it was not always possible to prepare depending on the circumstances of where and how she encountered a client. She recounted:

For example, when you meet with clients at night, when you go to a place you can’t say you have made preparations for the place you are heading to. But I make preparations when I find a client [...] I leave with the client, and the greatest preparation is informing your fellow [co-workers] where you are heading to [...] when you leave with a client what is important is that I took a private number and copied it in my phone in case the client shows any changes [and turns violent]. [...] The private number is at the back of the car you take before departing with a client without them noticing in case he does any cruel action towards you. -Tanzania, age 26

These narratives reveal that mobility contributed to uncertainty and insecurity among the participants in both settings. Paradoxically, mobility also promoted a sense of autonomy and agency among the women. Participants often reported feeling empowered to make their own choices related to sex work.

The second in vivo code, *I choose where I stay and everything is on my terms*, referred to an important perceived benefit of mobility that many of the participants across the two geographical settings experienced. A participant described how she was able to decide her own schedule when she traveled to Sosúa, a popular tourist beach town located in the northern province of Puerto Plata:

I am self-employed, and I have the freedom to figure out my own schedule when I travel to Sosúa. That’s why I travel, because of my economic situation. My goal is to get money. During the day I go to the beach, that is, many tourists go to the beach to swim and eat and there I can meet them. Then I go to the nightclub between twelve-thirty and one until three or four in the morning, sometimes five in the morning. -Dominican Republic, age 28

Similarly, another woman explained how she made decisions for herself related to the circumstances surrounding her travels:

On a normal day I stay in a small hotel, and I stay there because I feel good there. I decide the time I want to go to bed. I am in control. I get there early, I have my couple of clients that I call, and I tell them I’m going there, and they meet me when I want, and they are safe. I prefer the small hotel than going to a business like a bar where I have to waitress and then go out with the client once I am done. I was looking for strategies that would suit me, so I am doing it this way. I go without any commitment to the business owner. I have no commitment. I am self-employed and I am free to decide my plans. -Dominican Republic, age 37

A Tanzanian participant described the rationale in her decision-making process related to her sex work mobility:

Yes, that is when fishing season is at its peak in Mtera (located midway between Iringa and Dodoma on the border between the Iringa Region and the Dodoma Region), and the fishermen are having money. So since I know all the seasons, [...] I know right now when I go to Mtera I will make money, so I go there pretending to be a bartender, but my main job is to trade sex for money [...] I get my money and life continues [...] I can do it for a short time, but there are other options, I can choose to spend the whole night, what I do consider is that I get my money. -Tanzania, age 31

These participants’ experiences illuminated how mobility did not simply disrupt their lives but also engendered a sense of personal agency, whereby women in both settings made their own decisions and negotiated the terms related to their sex work with the goal of making money.

### Theme 2: Economic disruptors and benefits—financial insecurity versus profitability

The second theme is related to the economic disruptors and benefits that mobility created for Dominican and Tanzanian participants. The first in vivo code, *I make less money than expected sometimes when I travel*, referred to the economic disruptor of sex work mobility for both Tanzanian and Dominican women. They described how sometimes they end up making less money than expected because of exogenous factors, such as fewer clients than anticipated or low harvest yields in the rural areas. The diminished clientele resulting from clients’ economic struggles resulted in financial struggle. These factors had a negative domino effect on the livelihoods of participants in both settings.

A participant portrayed how she was not making as much money as she expected when she traveled for sex work in Tanzania and how this situation posed major challenges for her:

We have turbulent months sometimes. You will find that I have planned that I will get fifty thousand shillings (~$21.50) per day [...] then you go there and get back with twenty thousand shillings (~$8.50) a day. On the other side, you have the michezo (i.e., community savings group that you pay into as a member and offers loans to its members) where you have to contribute twenty-five thousand shillings (~$10.75), [...] and you have not eaten, you don’t have cooking oil, and you have not dressed. -Tanzania, age 27

In a similar vein, another participant contemplated whether she should stay in her specific location or continue with her travels to another destination. As she weighed the pros and cons, it became clear that the economic costs drove her decision to leave:

I went to Makambako (a medium-sized town and district in the Njombe Region of the Tanzanian Southern Highlands, located at the junction of two main roads between the cities of Njombe, Iringa, and Mbeya) to look for a job. Honestly, I did get there but the challenge was you stay overnight to work and the person you are working for deducts your money at the end of the day. You come to get paid maybe twenty thousand Tanzanian shillings (~$8.60). For me, what could twenty thousand do? Because I have not eaten, I have not put on clothes, money for my child [...] so I thought it doesn’t pay and I left. -Tanzania, age 28

Other participants in both Tanzania and in the Dominican Republic echoed that sex work mobility was not always profitable:

Through all that I have been through, I have not seen any kind of success or achievement. All the places that I go for work, be it the lake, or Dodoma, or anywhere else, the business wasn’t good. -Tanzania, age 34.

Similarly, a participant spoke to the unpredictable nature of encountering clients in a town she visited called Nagua in the northeastern Dominican Republic. Nagua’s economy relies heavily on the production of agricultural products, principally rice, coconuts, and cocoa bean.

There were not too many clients at my usual hotel when I went to Nagua. I ended up going to the nightclub and then I sat on a bench in the park waiting to see if I could get more clients. -Dominican Republic, age 50

Similarly, another participant in the DR shared how factors outside one’s control, like a poor harvest, directly impacted women’s livelihoods and contributed to economic insecurity.

I’m going to the rural area in Castillo (rural area in the north-central part of the island) for sex work, but it is hard now because there is no money; things are really bad there. Before I would stay a whole week, but the last time I left prematurely. I go to two businesses there, but the thing is that people there are economically dependent on the cacao and there is no cacao. Last year when there was a bad harvest, they lowered the market price too and that was terrible because this meant that the male clients could not pay me for my services. They did not have any cash and many of them were on drugs. The women offered sex for drugs or for very little money. *-*Dominican Republic, age 53

Across both geographical settings, women reported facing economic instability, whereby mobile sex work did not always bear fruit and in fact sometimes led to economic insecurity.

On the other hand, there was a powerful economic benefit of being mobile for sex work as it increased the likelihood of profitability for the women in the Dominican Republic and Tanzania. Thus, the second in vivo code, *I can make more money for me and my family when I travel*, referred to the economic benefit of mobile sex work. This second theme was linked directly to the first theme, which participants experienced a greater sense of personal autonomy and agency that contributed to not only economic survival but also economic independence as well. Regarding the first theme, participants mentioned making decisions about sex work mobility, including where to travel, based on their ability to make more money. A participant depicted how she decided to travel to the capital of Tanzania during a specific “season” for sex work, since she knew she would be likely to make more money:

In Dodoma, there are some seasons, like there is the grape season and when there is parliament session, in those times, money is available. So, I do know that at that particular time when I go there, I will come out with a lot of money, So, once we are there, we do our business, we trade sex for money. -Tanzania, age 36

She went on to share how she saved money by traveling because she set a limited budget and did not have bills to pay when she was mobile, reducing everyday expenses:

I see Dodoma as a place where I can earn money, there is a lot of money compared to Iringa. Here [at home] after a short time, they bring the water bill and the electricity bill so there are a lot of problems as opposed to there [at your travel destination]. Although you live in guest house when you travel, when you are there, you spend little money and you can set a limit that you will reserve fifty thousand (~$21.50 USD) for cell phone, you contribute ten thousand (~$4.30 USD) for michezo then five thousand (~$2.15 USD) for a guest and five thousand to spend on food. -Tanzania, age 33

The women described how they calculated the potential gains and losses of sex work mobility, and if the gains outweighed the losses, they traveled for sex work. One Dominican woman recognized the trade-off in her decision to be mobile for sex work; yet, she traveled to a large city at the eastern tip of the island because she made more money being mobile:

When I go to Higüey to make money to support myself and my daughter and her family who live with me, it is difficult. It is far away from my home, and I go for two to three months by myself. But I am able to make more money when I travel far away so I started to go in this direction. When I worked here in El Cibao (region located in the northern part of the Dominican Republic), it was easier but I was not making as much money. -Dominican Republic, age 34

As reflected in this quote, many participants described how being mobile allowed them to gain more economic independence. For some, this meant the possibility to invest in the future for themselves and their family members. One participant spoke about how the money she made through her mobile sex work allowed her to invest in other money-generating activities to increase her capital. Another woman articulated her future plans. She hoped to buy a plot of land and build a small house there for herself, her two daughters, and her grandmother:

When I go to the campo in Castillo (rural area in the north-central part of the DR) for ‘el chiripeo’ (sex work), I can make more money there. I like to invest it, like with 1000 pesos ($17 USD) I made, I buy Arizolín [cleaning products]. I negotiate a good price for the products and then I resell them. I can make extra money. -Dominican Republic, age 53

I am able to plan a future for myself and my family with the money from my ‘chiripeo’ (sex work) in Sosúa, a beach town where a lot of tourists go. I travel there for two weeks at a time to make money. With that money, I took the step of buying a lot to build a small house. I think that is a good start. This year I have plans to begin the construction of my house. I have two girls and I would not want to see them nor my mamá (grandmother) on the street. -Dominican Republic, age 30

These narratives reveal that the participants’ choice of destination was centered on the potential economic opportunity that the location provided. The next theme focused on how mobility impacted the HIV care and treatment of these participants who were all living with HIV.

### Theme 3: HIV care and treatment disruptors and benefits—disorder and interruptions versus strategy and social support

The third theme is related to the disruptors and benefits of mobility on HIV care and treatment among Dominican and Tanzanian participants. The first in vivo code, *I am inconsistent with my HIV appointments and medications when I travel*, referred to the disruptor of sex work mobility on the women’s HIV care and treatment. Both Dominican and Tanzanian women related how sometimes they ended up taking their medications inconsistently or stopped taking them altogether when they travel:

I was getting dizzy, and I was losing weight too, but I know that it was because of being mobile and how it affected my diet and the schedule of how I took my pills. [...] One day I took them in the morning, the next day I had to take them at night and so on [...] I ended up abandoning my HIV medication and treatment for four months. -Dominican Republic, age 39

When I travel for work, my schedule sometimes changes while I am there. I had to take the pill at one o’clock, and sometimes I didn’t take it thinking that I was going to come back home, but if they paid me to stay for two or three days more, I would not take the pill. On other occasions, I also run out of pills. These are the things when I am traveling that impede me from taking my pills consistently. -Dominican Republic, age 21

Similarly, the participants described how mobility contributed to missed doses of their ART medication:

I take them today then for about three days, I don’t take the medicine because sometimes when I’m travelling, I have forgotten to take the medications with me. -Tanzania, age 26

For instance, there are times that I drink when I am with a client, I will not take my medication [...] sometimes I rush and take a bus to meet with the client in a neighboring town […] when I go with the client, I do not end up returning and sometimes end up staying a week longer [...] and that is when I miss my medication or do not take it on time. -Tanzania, age 32

When asked, for example, if they would take a long-acting injectable form of ART if it were available, participants reported that they would prefer an injection over pills, due to the disruptions of mobility on their adherence to HIV medications.

Mobility also impacted the participants’ ability to adhere to some of their HIV care appointments:

Traveling sometimes interferes with my appointments for my HIV condition. Well, sometimes I’m not here in Santo Domingo, I have to take a trip for work, and I miss the day of the appointment. I’m in another city with a client and I cannot leave because he asked me to stay with him for a few extra days. -Dominican Republic, age 41

Across both contexts, participants shared their challenges with adhering to HIV care appointments and ART due to their mobility.

The second in vivo code in this theme highlighted the paradoxical nature of the impact of mobility on HIV care. Mobility contributed to participants often becoming more strategic and creative in their HIV care and treatment. Therefore, the second in vivo code was *I find ways to keep up with my HIV appointments and medications and rely on other FSWs when I travel*. When the women were proactively making decisions about their mobility, they considered how it would directly impact their HIV care and treatment and found creative and strategic ways not to interrupt their care. Participants shared experiences of how they scheduled their HIV appointments around their mobility plans to reduce or prevent interruptions in their HIV care:

With my appointments at the clinic, I schedule them for certain days. It can be Friday but not Monday. For example, I come to my appointment on Friday and then I catch the bus for my trip from the clinic. There is a bus stop near the clinic that I take. Mondays are not good because it would mean having to cut my trip short so I do not miss my appointment. -Dominican Republic, age 39

Other participants, in both geographical settings, recounted how they proactively calculated the amount of ART medication they need to obtain before travel to ensure they have enough:

If I am going to last five days on my trip, I take enough medication for eight or ten days just in case I need it if I stay longer. -Dominican Republic, age 37

Participants also spoke of their strategies to obtain a greater supply of their medications than what they needed before traveling to their destination:

In Mozambique, you are contracted for three months, so when you leave here and you have been given three bottles you inform them [doctors] that you are traveling somewhere and they add two more bottles so that it is five. -Tanzania, age 29

Mobile women living with HIV in both contexts demonstrated ingenuity in managing the HIV stigma by employing creative strategies to adhere to their HIV medications, without clients and others realizing the medication’s purpose:

When I travel for work, I put my HIV medications in another pill bottle like in my Omeprazole [used to treat certain stomach and esophagus problems] pill bottle in case people ask, ‘What is that?’ So, I carry my medications in that pill bottle and I take it when it is time to take it. -Dominican Republic, age 34

The participants also found inventive ways of hiding their ART medications when they were on the move. One woman discussed how she hid them in a tissue or in her handbag, while another took her medications in the bathroom or while the client is in the bathroom:

I never miss [my medication], even if I go away. If I go away, I can hide it in a tissue paper so when sleeping time arrives, I can act like I put something in my mouth discreetly. On other occasions, I say I am taking Sumatriptan [used to treat migraine headaches] because we usually use Sumatriptan so when someone asks me, I can tell them that I have a headache [...] This is what I do because I do not want to miss taking my medicine. -Tanzania, age 40

Moreover, they figured out ways to not miss their antiretroviral medications when they were mobile:

When I go on my trips for work, if I feel tired sometimes then I could easily forget to take my HIV medications. I decided to set an alarm. I set the alarm on my watch and on my cell phone. When the alarm rings, I can remember to take my medications. -Dominican Republic, age 41

Participants also discussed how they demonstrated solidarity by offering each other emotional and tangible forms of social support for continuity with their HIV care and treatment. In the example below, a participant referred to how women received and provided informational and resource support related to HIV care and treatment:

Many of us know each other and count on each other. I have had HIV for a longtime and some of the other women who travel here have not known their status for as long as me. After I meet them and get to know them a little bit, I share helpful information and resources about HIV with them. -Dominican Republic, age 28

Similarly, a woman described the support she received from a fellow FSW, Afaafa, who got her HIV medications for her before they traveled together to a town in Central Tanzania:

Afaafa is reminding me: ‘Did you remember that it is the day to go to Iringa?’ […] I will go and get your medicine for you. She goes and gets medicine for me before we travel…-Tanzania, age 37

Participants discussed how they sometimes shared their ART medications with one another when it was necessary, allowing them to continue with their HIV treatment:

When I went to Chunya [one of the seven districts of the Mbeya Region in Tanzania], I didn’t carry my [prescription] card with me. So, one sister had three bottles. I asked for one and she gave it to me. We knew each other. […] She said: ‘Don’t worry, I will give it to you. -Tanzania, age 40

Despite mobility being a source of disruption for HIV care and treatment among women, the participants’ narratives shed light on how it also created benefits in terms of personal agency and social support in their HIV care and treatment.

## Discussion

This in-depth qualitative study is the first to investigate perceptions, experiences, and influences of mobility on the lives of FSWs living with HIV across two distinct geographical settings. We found that mobility among this population in two geographic and epidemic sites is not a unidimensional and clear-cut process. By being mobile, Dominican and Tanzanian women living with HIV faced greater uncertainty, economic insecurity, and interruptions to HIV care and treatment; however, they also experienced benefits related to mobility including greater control, profitability, and social support for HIV care and treatment.

Through work-motivated mobility, women often exert their agency and economic independence. Instead of being limited to a certain location, participants in both geographical settings can strategize their mobility in a manner that benefits them. Many of the women interviewed describe how sex work-related mobility allows them to be more financially independent as well as empowered to make decisions regarding their work. This independence as a result of mobility sheds light on how the participants are entrepreneurial women who are helping to support their families, deconstructing the negative stereotypes often imposed upon them [27, 28].

Mobile sex work offers what Hall [21] refers to as a form of capital for some women, whereby it allows them to increase their income beyond what it would have been if they stayed in one location. This notion is consistent with our study findings, which reveal that the participants travel to work in more distant venues or environments that provide greater economic opportunity. Additionally, the participants across both settings rely on the unique forms of social support that mobility offers to ensure greater personal security and less interruptions with HIV care and treatment.

Our findings related to both the disruptors and benefits of sex work mobility are also in line with findings from other studies with women in other geographical settings such as China and India [10, 27]. For example, in Yu et al.’s study [10], mobile Chinese women demonstrate a strong sense of agency as they engage in sex work mobility despite adverse structural factors. This study also demonstrates the manner in which these women are creative, independent, and keenly attuned to growing and changing economic demands.

Our results also shed light on sex work mobility as a complex and nuanced phenomenon when considering the narratives that depict mobile FSWs not as a monolithic group but instead as women who have multifaceted lives and experiences [27, 28]. Often, the Dominican and Tanzanian women interviewed discussed how they are juggling multiple life roles, such as student, mother, daughter, and sister, in addition to multiple occupations ranging from informal sex work to formal sectors (e.g., pharmacist assistant in the specific case of a Dominican woman) as they navigate both the disruptions and benefits that mobility engenders.

Study findings have important implications for the development of intervention approaches that center the role of mobility and its influence on the lives, livelihoods, and HIV care and treatment trajectories of these women. For instance, findings related to how mobility contributes not simply to disruptions but to benefits provide important insights related to an intervention approach. Strengths-based approaches have evolved to challenge the more widely used deficit approaches. These approaches focus on enhancing the assets (at the individual and collective level) and resources (at the community/structural level) [29]. Adopting a strengths-based community empowerment approach aims to map the benefits of mobility (e.g., personal agency, social support) and leverage the existing individual and collective resiliencies and capabilities of women living with HIV. At the same time, this approach mitigates the disruptions mobility can create by being an effective approach tailored to meet the needs of this mobile population. Previous studies indicate that community empowerment approaches where FSWs drive the response to HIV and strategically engage partners to target socio-structural and environmental factors have a demonstrable impact on HIV prevention, treatment, and care outcomes [2, 30].

One potential element that may be used within a strengths-based, community empowerment approach for mobile women that would increase individual and collective agency and empowerment through the FSWs’ social networks may be an mHealth phone application [31–35]. This application would provide a virtual social network of FSWs who cross geographical boundaries. On the application, women could interact and offer peer support, providing a sense of social cohesion. Examples for the application’s capabilities could be that it tracks areas of security concerns through women reporting incidents, in addition to providing locations of proximal GBV and HIV treatment services. The women could have the ability to share information about local resources and in-person support networks, including peer leaders in communities where FSWs travel or are considering travel. This mHealth phone application would aim to empower women living with HIV by increasingly allowing them to assert more control over their working environments, make strategic decisions regarding their mobility plans and HIV care and treatment, employ creative strategies that mitigate potential disruptors to HIV care, and ultimately, shape their own lives.

Our findings point to the already established creative and effective strategies that women living with HIV in the Dominican Republic and Tanzania employ to manage the disruptions of mobility in their lives and HIV care and treatment. These strategies can be shared among other women as best practices when traveling for work. For example, the development of innovative strategies to adhere to their ART medication during mobility, such as alarms to remind themselves when to take the pills during their travels and changing the label of their medicine bottles to disguise the HIV medication when they travel, can be shared with other women. They are also able to sometimes schedule their HIV appointments around their mobility plans to reduce or prevent interruptions in their HIV care, obtain their ART at their travel destinations, or proactively calculate the amount of ART they need before they travel.

In addition, the findings have implications for existing HIV service provision. HIV services should be flexible in adapting to effectively meet the unique needs of this mobile population. Examples include mobile HIV treatment centers, virtual HIV support groups, more flexible hours for HIV care appointments to accommodate travel plans, and increased prescriptions for longer trips to avoid ART interruption [36–39]. Previous studies demonstrate that women are highly likely to use long-acting (LA) injectable ART, a potentially more convenient and private option for accessing ART [40–42]. Our study findings indicate that mobile FSWs in particular are a key population that may benefit from and be interested in LA injectable ART. It would address the oral ART adherence interruptions and HIV-related stigma mentioned by the study participants given that it requires monthly or every 2-month injections, eliminating the need for daily pills and strategies to hide the pills or reminders to take them. These structural changes can enable women to financially benefit from sex work mobility while also effectively sustaining their HIV care and treatment during their travels.

This study has limitations and strengths. Our initial recruitment of the larger cohort of Dominican participants from which we derived our qualitative sub-cohorts was predominantly comprised of women who were active in our DR partner sex worker organization and, for the Tanzanian cohort, those who were active in specific venues or areas on particular days and times [2]. The experiences of these participants may be different from those who are not active in our sex worker partner organization in the DR or in specific venues/areas in Tanzania; thus, their mobility experiences may not be transferable to other mobile women in the DR and Tanzania. In addition, there may have been recall bias given that participants were asked to speak about their past mobility experiences. Lastly, given that these interviews were conducted in Spanish and Swahili, respectively, there may have been linguistic and cultural equivalence challenges when translating and interpreting the data.

Despite these limitations, the study also had important strengths, including the use of a rigorous qualitative methodology allowing us to gather comprehensive information to improve our understanding of participants’ perceptions and experiences related to sex work mobility. The in-depth “thick descriptions” [43] elicited a deeper understanding of the nuanced and paradoxical nature of sex work mobility. Thematic analysis as the main analytic method was also a strength of this study as it facilitated the identification of salient themes and subthemes across the interviews. In addition, we explored the phenomenon of mobility across two geographic and epidemic settings employing an intra- and inter-comparison approach of the data, which allowed us to identify patterns within and across the two contexts. Interviews were also conducted over two different time periods which allowed us to go more in-depth and follow up on themes with participants. Another strength of this study involved the inclusion of community-based study partners with extensive experience and key networks, making it highly feasible to reach and retain participants. Moreover, we used independent interview coders to ensure the validity of our findings.

## Conclusions

The findings shed light on the paradoxical experiences of FSWs living with HIV whose lives both benefit from and are disrupted by mobility. This information is critical in informing our efforts to successfully respond to the unique needs of this population. Programs tailored to meet the needs of this mobile population are needed to effectively mitigate disruptors of sex-related mobility while maximizing benefits for this population. Doing so would enable women to enhance their agency in making their own decisions related to sex work mobility and to economically benefit from mobile sex work in a safe environment, without interruption to their HIV care and treatment. Future studies are warranted to further examine the impact of mobility among FSWs living with HIV in other geographic and epidemic settings, elucidate whether there are similar patterns across these populations, and evaluate programs tailored to their specific needs.

## Supplementary Information

**Additional file 1. **Interview guide.

**Additional file 2. **COREQ checklist.

## Data Availability

The dataset consisting of all the participant quotes and the memos analyzed during the current study are available from the corresponding author, Dr. Maria De Jesus, via an email request (dejesus@american.edu) due to adherence to privacy and confidentiality agreements.
