# Supplementary material for: Mobility paradoxes: disruptors, benefits, and agency among mobile female sex workers living with HIV in the Dominican Republic and Tanzania
Source: BMC Glob Public Health. 2024 Jan 16;2:5. doi: 10.1186/s44263-023-00032-3 (PMC11622933; doi:10.1186/s44263-023-00032-3)
Supplement: Supplementary file 1 — Additional file 1. Interview guide. [file 44263_2023_32_MOESM1_ESM.docx]

Interview Guide

Module on Mobility

1. Where do you consider “home?” Is it in [Iringa/Santo Domingo] or outside of [Iringa/Santo Domingo]? Why?
2. Think about all of the different places you typically go in [Iringa/Santo Domingo] and outside of [Iringa/Santo Domingo]. Where do you go? [Free-listing activity]
   1. Probes:
      1. **When** do you typically go? Is there a particular season or time of year?
      2. **Why** do you go there? If for work, what type of work do you do there? Do you exchange sex for money when you travel there? [Note: to adapt language to incorporate locally relevant language related to sex work.]
3. Tell me about a typical trip you’ve made outside of [Iringa/Santo Domingo].
   1. Probes:
      1. **Where** did you go? How long were you gone?
      2. **Preparations**: Did you make any arrangements before you traveled outside of [Iringa/Santo Domingo]? [e.g. places to stay, transportation, food, time off of work, care for children, telling friends/family, etc.]
      3. **Trip**: What was the trip like?
         1. How did you get there? How reliable was it? Did you travel with anyone? Who?
         2. Did you have any inconveniences or difficulties? Any specific dangers/fears?
         3. The next question is a sensitive one: Describe any violence you may have experienced before, during, and/or after you got to your destination.
      4. **Why** did you go there?
         1. Did you stop anywhere else along the way? Describe.
         2. Did you exchange sex for money when you traveled there? [Note: to adapt language to incorporate locally relevant language related to sex work; Probe on direct and/or indirect sex-related activities; e.g., working at bar, salon, etc.]
         3. How are conditions there different compared to your regular work environment in [Iringa/Santo Domingo]? [Probe on circumstances surrounding sex work and type of clientele]
         4. Describe experiences with alcohol and/or other drugs.
      5. **Needs**: What were your greatest needs while you were traveling?
      6. **Care-seeking/adherence**: Did you face any problem accessing HIV services while you were outside of [Iringa/Santo Domingo]? Or taking your medications for HIV while you were outside of [Iringa/Santo Domingo]? How could services be improved to prevent these problems?
      7. **Social relationships**: Did you have any friends/family where you went? Who?
         1. Did you feel like you had support there? What kinds of support (e.g., emotional, sense of community, resources, organizations)? From whom?
         2. Did you contact anyone while you were traveling? Who? How often? Why? Describe any challenges you may have had when contacting them. If contacted using a cell phone, ask: Do you use your own phone or someone else’s? If not your own, why do you not have your own cell phone?
      8. **Cell phone use**: For what purpose do you mainly use a cell phone? Do you use a cell phone when you travel? Could you receive text messages or phone calls while traveling? What do you use it for when you travel? Is that different from when you are in [Iringa/Santo Domingo]? How so?
4. There are many programs working in [Iringa/Santo Domingo] to improve people’s health. How could activities be designed for women like you who travel outside of [Iringa/Santo Domingo] frequently?
5. Would you want programs to be able to reach you when you are traveling? In your opinion, how could they best reach you when you are outside [Iringa/Santo Domingo]?
   1. Have you ever called a call/help center [Note: to adapt language to incorporate locally relevant language] while traveling? What services did you receive? What other services could a call/help center offer that you think would be beneficial for you when traveling?
   2. If someone were to call or text you while you were traveling, what resources or services in particular could they offer that you think would be beneficial for you when traveling?
