# Supplementary material for: Mobility paradoxes: disruptors, benefits, and agency among mobile female sex workers living with HIV in the Dominican Republic and Tanzania
Source: BMC Glob Public Health. 2024 Jan 16;2:5. doi: 10.1186/s44263-023-00032-3 (PMC11622933; doi:10.1186/s44263-023-00032-3)
Supplement: Supplementary file 2 — Additional file 2. COREQ checklist. [file 44263_2023_32_MOESM2_ESM.docx]

Consolidated criteria for reporting qualitative studies (COREQ): 32-item checklist

| **No** | **Item** | **Guide questions/description** | **Reported on Page No.** |
| --- | --- | --- | --- |
| **Domain 1: Research team and reflexivity** |  |  |  |
| Personal Characteristics |  |  |  |
| 1. | Interviewer/facilitator | Four trained interviewers conducted the interviews. | Page 7 |
| 2. | Credentials | One MD, others trained on interview skills | Page 7 |
| 3. | Occupation | Doctor, peer navigator | Page 7 |
| 4. | Gender | Female | Page 7 |
| 5. | Experience and training | Extensive research experience including with data collection and analysis | Page 7 |
| Relationship with participants |  |  |  |
| 6. | Relationship established | A relationship with community partner organizations | Pages 6-7 |
| 7. | Participant knowledge of the interviewer | The participants were informed about the reasons for doing the research | Page 7 |
| 8. | Interviewer characteristics | Four trained interviewers | Page 7 |
| **Domain 2: study design** |  |  |  |
| Theoretical framework |  |  |  |
| 9. | Methodological orientation and Theory | A thematic analysis methodological approach and a social determinants framework guided the study. | Page 8 |
| Participant selection |  |  |  |
| 10. | Sampling | This study focuses on the qualitative data from the second round of interviews with a subset of cohort participants in each country (n=12 Dominicans and n=12 Tanzanians), who had reported traveling outside of Santo Domingo/Iringa in the last 6 months in the quantitative survey. | Page 7 |
| 11. | Method of approach | Participants were approached face-to-face. | Page 7 |
| 12. | Sample size | 24 | Page 7 |
| 13. | Non-participation | 0 | Page 7 |
| Setting |  |  |  |
| 14. | Setting of data collection | Interviews were conducted in clinical sites in Santo Domingo and Iringa, respectively. | Page 7 |
| 15. | Presence of non-participants | No one else was present besides the participants and researchers. | Page 7 |
| 16. | Description of sample | Participants across geographic settings ranged from 20 to 52 years old. On average, the Dominican participants were older (mean=40) compared to the Tanzanian participants (mean=28). Most of the participants in Tanzania and approximately half of the Dominican participants were single or not currently living with a partner. All participants in the sample had children, with a mean number of 2 and all lived in either Santo Domingo (capital of the DR) or Iringa (city in central Tanzania), respectively. Education levels were low with all the participants from the Dominican Republic having primary-level education and those from Tanzania having either primary-level education (7) or some secondary schooling (5). The participants in Tanzania had been engaging in sex work for an average of 8 years mostly in modern bars, while Dominican participants had been engaging in sex work for a much longer duration (21 years) in different venues (i.e., on the street or in a sex establishment) or independently. The frequency of sex work varied across both settings and the length of stay at their travel destination ranged from one day to three months. | Pages 9-10 |
| Data collection |  |  |  |
| 17. | Interview guide | The interviewers used an interview guide (see Additional file 1). | Page 7 |
| 18. | Repeat interviews | Yes, repeat interviews were carried out (n=24). | Page 7 |
| 19. | Audio/visual recording | The researcher used an audio recording to collect the data. | Page 8 |
| 20. | Field notes | The researcher used memo writing throughout the coding process to document emerging themes and observations about the data and our thoughts about the significance and relationships of codes to one another, as well as to assist with data reduction and interpretation. | Page 8 |
| 21. | Duration | The duration of the interviews was 1-1.5 hours. | Page 7 |
| 22. | Data saturation | We did not discuss data saturation. | Page 6 |
| 23. | Transcripts returned | Transcripts were not returned to participants for comment and/or correction. | Page 8 |
| **Domain 3: analysis and findings**z |  |  |  |
| Data analysis |  |  |  |
| 24. | Number of data coders | Two data coders coded the data. | Page 8 |
| 25. | Description of the coding tree | We did not provide a description of the coding tree as we did not use one. For this study, we developed a codebook based on the themes emerging from the data. Using our memos, we then synthesized the coding output across key domains, identified major themes, and listed in vivo codes under major themes. | Page 9 |
| 26. | Derivation of themes | Themes were derived from the data. The thematic analysis approach was an iterative, data-driven, and inductive process. We used memo writing throughout the coding process to document emerging themes. | Page 9 |
| 27. | Software | None |  |
| 28. | Participant checking | Participants did not provide feedback on the findings. Peer navigators provided feedback. |  |
| Reporting |  |  |  |
| 29. | Quotations presented | Participant quotations were presented to illustrate the and each quotation was identified with the participant’s age. | Pages 11-21 |
| 30. | Data and findings consistent | There was consistency between the data presented and the findings. | Page 22 |
| 31. | Clarity of major themes | Major themes were clearly presented in the findings. | Pages 11-21 |
| 32. | Clarity of minor themes | There is a description of minor themes and nuances within particular major themes. | Pages 11-21 |
